# Strongly Lacunary Ward Continuity in 2-Normed Spaces

**DOI:** 10.1155/2014/479679

**Published:** 2014-06-23

**Authors:** Hüseyin Çakalli, Sibel Ersan

**Affiliations:** ^1^Department of Mathematics, Maltepe University, Marmara Eğitim Köyü, 34857 İstanbul, Turkey; ^2^Faculty of Engineering and Natural Sciences, Maltepe University, Marmara Eğitim Köyü, 34857 İstanbul, Turkey

## Abstract

A function *f* defined on a subset *E* of a 2-normed space *X* is strongly lacunary ward continuous if it preserves strongly lacunary quasi-Cauchy sequences of points in *E*; that is, (*f*(*x*_*k*_)) is a strongly lacunary quasi-Cauchy sequence whenever (*x*_*k*_) is strongly lacunary quasi-Cauchy. In this paper, not only strongly lacunary ward continuity, but also some other kinds of continuities are investigated in 2-normed spaces.

## 1. Introduction

Menger [1] introduced a notion called a generalized metric in 1928, and ten years later Vulich [2] defined a notion of a higher dimensional norm in linear spaces. Unfortunately, these studies had been neglected by many analysts for a long time. The concept of a 2-normed space was developed by Gähler in the middle of 1960s [3–5]. Since then, Mashadi [6], Gurdal [7], Mazaheri and Kazemi [8], and Sahiner [9] have studied this concept and obtained various results.

Using the main idea in the definition of sequential continuity, many kinds of continuities were introduced and investigated, and not all but some of them are in [10–13]. The concept of *N*_*θ*_-convergence was introduced in [14] and further studied in [15]. Strongly lacunary ward continuity of a real function was introduced by Cakalli in [11] as named *N*_*θ*_-ward continuity and further studied in [16].

The aim of this paper is to investigate strongly lacunary ward continuity in 2-normed spaces and prove interesting theorems.

## 2. Preliminaries

Throughout this paper, *N* and *R* will denote the set of all positive integers and the set of all real numbers, respectively. Now we recall the definition of a two-normed space. Let *X* be a real linear space with dim⁡*X* > 1 and ||·, ·|| : *X* × *X* → *R* a function. Then (*X*, ||·, ·||) is called a linear 2-normed space if (i) ||*x*, *y*|| = 0⇔*x* and *y* are linearly dependent, (ii) ||*x*, *y*|| = ||*y*, *x*||, (iii) ||*αx*, *y*|| = |*α*|||*x*, *y*||, and (iv) ||*x*, *y* + *z*|| ≤ ||*x*, *y*|| + ||*x*, *z*|| for *α* ∈ *R* and *x*, *y*, *z* ∈ *X*. The function ||·, ·|| is called a 2-norm on *X*. Observe that in any 2-normed space (*X*, ||·, ·||) we have that ||·, ·|| is nonnegative, ||*x* − *z*, *x* − *y*|| = ||*x* − *z*, *y* − *z*||, and ||*x*, *y* + *αx*|| = ||*x*, *y*|| for all *x*, *y* ∈ *X*, *α* ∈ *R*. Throughout this paper by *X* we will mean a 2-normed space with a two-norm ||·, ·||. As an example of a 2-normed space we may take *X* = *R* ^2^ being equipped with the 2-norm

(1) $$\begin{array}{c} ||x_{1},x_{2}||=\text{abs}\left(\left|\begin{array}{cc} x_{11} & x_{12}\\ x_{21} & x_{22} \end{array}\right|\right), \end{array}$$

where *x*_1_ = (*x*_11_, *x*_12_), *x*_2_ = (*x*_21_, *x*_22_).

A sequence (*x*_*n*_) of points in *X* is said to converge to a point *L* of *X* in the 2-normed space *X* if lim⁡_*n*→*∞*_||*x*_*n*_ − *L*, *z*|| = 0 for every *z* ∈ *X*. This is denoted by lim⁡_*n*→*∞*_||*x*_*n*_, *z*|| = ||*L*, *z*||. A sequence (*x*_*n*_) of points in *X* is said to be a Cauchy sequence with respect to the 2-norm if lim⁡_*n*,*m*→*∞*_||*x*_*n*_ − *x*_*m*_, *z*|| = 0 for every *z* ∈ *X*. A sequence of functions (*f*_*n*_) is said to be uniformly convergent to a function *f* on a subset *E* of *X* if for each *ε* > 0, an integer *N* can be found such that ||*f*_*n*_(*x*) − *f*(*x*), *z*|| < *ε* for *n* ≥ *N* and for all *x*, *z* ∈ *X* [17]. A lacunary sequence *θ* = (*k*_*r*_) is an increasing sequence of positive integers such that *k*_0_ = 0 and *h*_*r*_ = *k*_*r*_ − *k*_*r*−1_ → *∞* as *r* → *∞*. The intervals determined by *θ* will be denoted by *I*_*r*_ = (*k*_*r*−1_, *k*_*r*_], and the ratio *k*_*r*_/*k*_*r*−1_ will be abbreviated by *q*_*r*_. A sequence (*x*_*k*_) of points in *X* is called strongly lacunary convergent, or *N*_*θ*_-convergent to an element *L* of *X* if

(2) $$\begin{array}{c} \underset{r\to \infty }{\lim}\frac{1}{h_{r}}\underset{k\in I_{r}}{\sum }||x_{k}-L,z||=0, \end{array}$$

for every *z* ∈ *X* and it is denoted by *N*_*θ*_ − lim⁡_*k*→*∞*_||*x*_*k*_, *z*|| = ||*L*, *z*|| for every *z* ∈ *X* [9]. Throughout the paper we will use the word “*N*_*θ*_” instead of “strongly lacunary” and assume that liminf⁡_*r*_ *q*_*r*_ > 1.

## 3. Results

The set of seminorms {*p*_*z*_} defined by *p*_*z*_(*x*) = ||*x*, *z*||, ∀*x*, for each *z* ∈ *X*, forms a locally convex topological vector space, and the topology formed by this family of seminorms gives the required topology on *X*. Since dim⁡*X* ≥ 2, for each *x* ∈ *X* there exists a *y* ∈ *X* such that *x* and *y* are linearly independent, and hence by (*i*), *p*_*y*_(*x*) = ||*x*, *y*|| ≠ 0. Thus the locally convex topological vector space induced by the set {*p*_*z*_ : *z* ∈ *X*} of seminorms is Hausdorff so *X* is a Hausdorff space [18]. In this section, we investigate the concepts of a strongly lacunary quasi-Cauchy sequence and strongly lacunary ward continuity of a function on a 2-normed space.

**Definition 1 .**

A subset *E* of *X* is called *N*_*θ*_-sequentially compact if any sequence of points in *E* has an *N*_*θ*_-convergent sequence with an *N*_*θ*_-limit in *E*.

We note that union of two *N*_*θ*_-sequentially compact subsets of *X* is *N*_*θ*_-sequentially compact, intersection of any *N*_*θ*_-sequentially compact subsets is *N*_*θ*_-sequentially compact, any compact subset of *X* is *N*_*θ*_-sequentially compact, and any finite subset of *X* is *N*_*θ*_-sequentially compact. Sum of *N*_*θ*_-sequentially compact subsets of *X* is *N*_*θ*_-sequentially compact where sum of two subsets *A* and *B* is defined as *A* + *B* = {*a* + *b* : *a* ∈ *A*, *b* ∈ *B*}.

**Definition 2 .**

A function *f* defined on a subset *E* of *X* is said to be strongly lacunary sequentially continuous or *N*_*θ*_-sequentially continuous at a point *x*_0_ of *E* if (*f*(*x*_*k*_)) is an *N*_*θ*_-convergent sequence to *f*(*x*_0_) whenever (*x*_*k*_) is an *N*_*θ*_-convergent to *x*_0_ sequence of points in *E*. If *f* is strongly lacunary sequentially continuous at every point of *E*, then it is said to be strongly lacunary sequentially continuous on *E*.

If a function *f* defined on a subset *E* of *X* is lacunary statistically sequentially continuous at a point *x*_0_, then (*f*(*x*_*k*_)) is an *N*_*θ*_-convergent sequence with *N*_*θ*_ − lim⁡||*f*(*x*_*k*_), *z*|| = ||*f*(*x*_0_), *z*|| for every *z* ∈ *X* whenever (*x*_*k*_) is an *N*_*θ*_-convergent sequence with *N*_*θ*_ − lim⁡||*x*_*k*_, *z*|| = ||*x*_0_, *z*|| for every *z* ∈ *X*. We see that a function *f* defined on a subset *E* of *X* is strongly lacunary sequentially continuous if and only if it preserves strongly lacunary convergent sequences without stating limit of the sequence. We note that sum of two *N*_*θ*_-sequentially continuous functions at a point *x*_0_ of *X* is *N*_*θ*_-sequentially continuous at *x*_0_, and composite of two *N*_*θ*_-sequentially continuous functions at a point *x*_0_ of *X* is *N*_*θ*_-sequentially continuous at *x*_0_. In the classical case, that is in the single normed case, it is known that uniform limit of sequentially continuous function is sequentially continuous; now we see that it is also true that not only uniform limit of sequentially continuous function is sequentially continuous, but also uniform limit of *N*_*θ*_-sequentially continuous function is *N*_*θ*_-sequentially continuous in 2-normed spaces. Now we give the latter in the following.

**Theorem 3 .**

Uniform limit of *N*_*θ*_-sequentially continuous functions is *N*_*θ*_-sequentially continuous.

**Proof**

Let (*f*_*k*_) be a uniformly convergent sequence of each term defined on a subset *E* of *X* with uniform limit *f* and let (*x*_*k*_) be any *N*_*θ*_-convergent sequence of points in *E* with *N*_*θ*_ − lim⁡||*x*_*k*_, *z*|| = ||*x*, *z*|| for every *z* ∈ *X*. Take any *ε* > 0. By uniform convergence of (*f*_*k*_), there exists an *n*_1_ ∈ *N* such that ||*f*(*x*) − *f*_*k*_(*x*), *z*|| < *ε*/3 for *k* ≥ *n*_1_ and every *x* ∈ *E* and *z* ∈ *X*. Hence,

(3) $$\begin{array}{c} \frac{1}{h_{r}}\underset{k\in I_{r}}{\sum }||f\left(x\right)-f_{k}\left(x\right),z||<\frac{1}{h_{r}}\left(k_{r}-k_{r-1}\right)\frac{\varepsilon }{3}=\frac{\varepsilon }{3}, \end{array}$$

for *r* ≥ *n*_1_ and every *x* ∈ *E* and *z* ∈ *X*. As *f*_*n*_1__ is *N*_*θ*_-sequentially continuous on *E*, there exists an *n*_2_ ∈ *N* such that, for *r* ≥ *n*_2_,

(4) $$\begin{array}{c} \frac{1}{h_{r}}\underset{k\in I_{r}}{\sum }||f_{n_{1}}\left(x\right)-f_{n_{1}}\left(x_{k}\right),z||<\frac{\varepsilon }{3}, \end{array}$$

for every *z* ∈ *X*. Now write *n*_0_ = max⁡{*n*_1_, *n*_2_}. Thus for *r* ≥ *n*_0_ we have

(5) 1hr∑k∈Ir||f(x)−f(xk),z|| ≤1hr∑k∈Ir||vk(x),z||+1hr∑k∈Ir||fn1(x)−fn1(xk),z||  +1hr∑k∈Ir||fn1(xk)−f(xk),z|| <ε3+ε3+ε3=ε,

where *v*_*k*_(*x*) = *f*(*x*) − *f*_*n*_1__(*x*) for every *k* ∈ *N*. Hence

(6) $$\begin{array}{c} \underset{r\to \infty }{\lim}\frac{1}{h_{r}}\underset{k\in I_{r}}{\sum }||f\left(x\right)-f\left(x_{k}\right),z||=0. \end{array}$$

This completes the proof of the theorem.

**Theorem 4 .**

*N*_*θ*_-sequentially continuous image of any *N*_*θ*_-sequentially compact subset of *X* is *N*_*θ*_-sequentially compact.

**Proof**

Assume that *f* is an *N*_*θ*_-sequentially continuous function on a subset *E* of *X* and *A* is an *N*_*θ*_-sequentially compact subset of *E*. Let (*f*(*x*_*n*_)) be any sequence of points in *f*(*A*) where *x*_*n*_ ∈ *A* for each positive integer *n*. *N*_*θ*_-sequentially compactness of *A* implies that there is a subsequence (*γ*_*k*_) = (*x*_*n*_*k*__) of (*x*_*n*_) with *N*_*θ*_ − lim⁡_*k*→*∞*_||*γ*_*k*_, *z*|| = ||*l*, *z*|| for every *z* ∈ *E*. Write (*t*_*k*_) = (*f*(*γ*_*k*_)). As *f* is *N*_*θ*_-sequentially continuous, (*f*(*γ*_*k*_)) is *N*_*θ*_-sequentially convergent which is a subsequence of the sequence (*f*(*x*_*n*_)) with *N*_*θ*_ − lim⁡_*k*→*∞*_||*t*_*k*_, *z*|| = ||*l*, *z*|| for ∀*z* ∈ *E*. This completes the proof of the theorem.

The concept of a quasi-Cauchy sequence in a 2-normed space was studied in [19]. Now we give the following definition of an *N*_*θ*_-quasi-Cauchy sequence.

**Definition 5 .**

A sequence (*x*_*n*_) of points in a subset *E* of *X* is called *N*_*θ*_-quasi-Cauchy if (Δ*x*_*k*_) is *N*_*θ*_-convergent to 0, that is,

(7) $$\begin{array}{c} N_{\theta }-\underset{k\to \infty }{\lim}||\Delta x_{k},z||=0, \end{array}$$

where Δ*x*_*k*_ = *x*_*k*+1_ − *x*_*k*_.

We note that any quasi-Cauchy sequence is *N*_*θ*_-quasi-Cauchy, so any convergent sequence is *N*_*θ*_-quasi-Cauchy in *X*. Any Cauchy sequence is *N*_*θ*_-quasi-Cauchy, but the converse is not always true. However the converse is not always true, that is, there are *N*_*θ*_-quasi-Cauchy sequences which are not convergent. Sum of two *N*_*θ*_-quasi-Cauchy sequences is *N*_*θ*_-quasi-Cauchy. Subsequence of an *N*_*θ*_-quasi-Cauchy sequence needs not be *N*_*θ*_-quasi-Cauchy. Now we give the definition of *N*_*θ*_-ward compactness of a subset of *X*.

**Definition 6 .**

A subset *E* of *X* is called *N*_*θ*_-ward compact if any sequence of points in *E* has an *N*_*θ*_-quasi-Cauchy subsequence.

Union of two *N*_*θ*_-ward compact subset of *X* is *N*_*θ*_-ward compact, intersection of any *N*_*θ*_-ward compact subsets is *N*_*θ*_-ward compact, sum of two *N*_*θ*_-ward compact subset of *X* is *N*_*θ*_-ward compact, and any finite subset of *X* is *N*_*θ*_-ward compact.

**Definition 7 .**

A function defined on a subset *E* of *X* is called *N*_*θ*_-ward continuous if it preserves *N*_*θ*_-quasi-Cauchy sequences, that is, (*f*(*x*_*k*_)) is an *N*_*θ*_-quasi-Cauchy sequence whenever (*x*_*k*_) is.

We note that a composite of two *N*_*θ*_-ward continuous functions is *N*_*θ*_-ward continuous, and sum of two *N*_*θ*_-ward continuous functions is *N*_*θ*_-ward continuous.

**Theorem 8 .**

*N*_*θ*_-ward continuous image of any *N*_*θ*_-ward compact subset of *X* is *N*_*θ*_-ward compact.

**Proof**

Assume that *f* is an *N*_*θ*_-ward continuous function on a subset *E* of *X* and *E* is a *N*_*θ*_-ward compact subset of *A*. Let (*f*(*x*_*n*_)) be any sequence of points in *f*(*E*) where *x*_*n*_ ∈ *E* for each positive integer *n*. *N*_*θ*_-ward compactness of *E* implies that there is a subsequence (*γ*_*k*_) = (*x*_*n*_*k*__) of (*x*_*n*_) with *N*_*θ*_ − lim⁡_*k*→*∞*_||Δ*γ*_*k*_, *z*|| = 0 for every *z* ∈ *E*. Write (*t*_*k*_) = (*f*(*γ*_*k*_)). As *f* is *N*_*θ*_-ward continuous, (*f*(*γ*_*k*_)) is *N*_*θ*_-quasi-Cauchy which is a subsequence of the sequence (*f*(*x*_*n*_)) with *N*_*θ*_ − lim⁡_*k*→*∞*_||Δ*t*_*k*_, *z*|| = 0 for ∀*z* ∈ *E*. This completes the proof of the theorem.

**Corollary 9 .**

*N*_*θ*_-ward continuous image of any compact subset of *X* is *N*_*θ*_-ward compact.

**Proof**

The proof follows from the preceding theorem.

A function *f* defined on a subset *E* of *X* is sequentially continuous at *x*_0_, if for any sequence (*x*_*n*_) of points in *E* converging to *x*_0_, we have (*f*(*x*_*n*_)) converges to *f*(*x*_0_). *f* is sequentially continuous on *E* if it is sequentially continuous at every point of *E* (see [19] for the infinite dimensional case and [20] for the finite dimensional case).

Concerning *N*_*θ*_-quasi-Cauchy sequences, *N*_*θ*_ convergent sequences, and convergent sequences the problem arises to investigate the following types of continuity of functions on *X*. In the following *N*_*θ*_, Δ*N*_*θ*_, and *c* will denote the set of *N*_*θ*_-convergent sequence, the set of all *N*_*θ*_-quasi-Cauchy sequences, and the set of convergent sequences of points in *X*, respectively:

1. (*x*_*k*_) ∈ Δ*N*_*θ*_⇒(*f*(*x*_*k*_)) ∈ Δ*N*_*θ*_,
2. (*x*_*k*_) ∈ Δ*N*_*θ*_⇒(*f*(*x*_*k*_)) ∈ *c*,
3. (*x*_*k*_) ∈ *c*⇒(*f*(*x*_*k*_)) ∈ *c*,
4. (*x*_*k*_) ∈ *c*⇒(*f*(*x*_*k*_)) ∈ Δ*N*_*θ*_,
5. (*x*_*k*_) ∈ *N*_*θ*_⇒(*f*(*x*_*k*_)) ∈ *N*_*θ*_,
6. (*x*_*k*_) ∈ Δ*N*_*θ*_⇒(*f*(*x*_*n*_))  is quasi-Cauchy,
7. (*x*_*k*_) ∈ Δ*N*_*θ*_⇒(*f*(*x*_*n*_)) ∈ *N*_*θ*_.

We see that (1) is *N*_*θ*_-ward continuity of *f*, (5) is *N*_*θ*_-sequentially continuity of *f*, and (3) is the ordinary sequential continuity of *f*. It is easy to see that (2) implies (1), and (1) does not imply (2); and (1) implies (4), and (4) does not imply (1); (2) implies (3), and (3) does not imply (2). (6) implies (1), but (1) does not imply (6) and (7) implies (1), but (1) does not imply (7). Now we give that the implication (1) implies (5), that is, any *N*_*θ*_-ward continuous function is *N*_*θ*_-sequentially continuous.

**Theorem 10 .**

If *f* is *N*_*θ*_-ward continuous function on a subset *E* of *X*, then it is *N*_*θ*_-sequentially continuous on *E*.

**Proof**

Assume that *f* is an *N*_*θ*_-ward continuous function on a subset *E* of *X*. Let (*x*_*n*_) be any *N*_*θ*_-convergent sequence in *A* with *N*_*θ*_ − lim⁡_*k*→*∞*_||*x*_*k*_, *z*|| = ||*x*_0_, *z*|| for all *z* ∈ *X*. Then the sequence *x* = (*x*_*n*_) defined by

(8) $$\begin{array}{c} x_{n}=\{\begin{array}{cc} x_{k}, & \text{if}\;\;n=2k-1\;\text{for}\;\text{a}\;\text{positive}\;\text{integer}\;k\\ x_{0}, & \text{if}\;\;n\;\text{is}\;\text{even} \end{array} \end{array}$$

is also *N*_*θ*_-convergent to *x*_0_. Hence it is *N*_*θ*_-quasi-Cauchy sequence. As *f* is *N*_*θ*_-ward continuous on *E*, the transformed sequence (*y*_*n*_) obtained by

(9) $$\begin{array}{c} y_{n}=\{\begin{array}{cc} f\left(x_{k}\right), & \text{if}\;\;n=2k-1\;\text{for}\;\text{a}\;\text{positive}\;\text{integer}\;k\\ f\left(x_{0}\right), & \text{if}\;\;n\;\text{is}\;\text{even} \end{array} \end{array}$$

is also *N*_*θ*_-quasi-Cauchy. Thus,

(10) $$\begin{array}{c} \underset{r\to \infty }{\lim}\frac{1}{h_{r}}\underset{k\in I_{r}}{\sum }||f\left(x_{k}\right)-f\left(x_{0}\right),z||=0 \end{array}$$

for all *z* ∈ *X*. It follows from this that the sequence (*f*(*x*_*k*_)) is *N*_*θ*_ convergent to (*f*(*x*_0_)).

The converse of this theorem is not valid in general, a counterexample can be easily constructed via the function *f*(*x*, *y*) = (*x* ^2^, *y* ^2^) on the 2-normed space *R* ^2^ with the usual 2-norm.

**Theorem 11 .**

If (*f*_*n*_) is a sequence of *N*_*θ*_-ward continuous functions on a subset *E* of *X* and (*f*_*n*_) is uniformly convergent to a function *f*, then *f* is *N*_*θ*_-ward continuous on *E*.

**Proof**

Let (*x*_*k*_) be any *N*_*θ*_-quasi-Cauchy sequence of points in *E*, and let *ε* be any positive real number. By uniform convergence of (*f*_*k*_), there exists an *n*_1_ ∈ *N* such that ||*f*(*x*) − *f*_*k*_(*x*), *z*|| < *ε*/3 for *k* ≥ *n*_1_ and every *x* ∈ *E* and *z* ∈ *X*. Hence,

(11) $$\begin{array}{c} \frac{1}{h_{r}}\underset{k\in I_{r}}{\sum }||f\left(x\right)-f_{k}\left(x\right),z||<\frac{1}{h_{r}}\left(k_{r}-k_{r-1}\right)\frac{\varepsilon }{3}=\frac{\varepsilon }{3}, \end{array}$$

for *r* ≥ *n*_1_ and every *x* ∈ *E* and *z* ∈ *X*. As *f*_*n*_1__ is *N*_*θ*_-ward continuous on *E*, there exists an *n*_2_ ∈ *N* such that, for *r* ≥ *n*_2_,

(12) $$\begin{array}{c} \frac{1}{h_{r}}\underset{k\in I_{r}}{\sum }||f_{n_{1}}\left(x_{k+1}\right)-f_{n_{1}}\left(x_{k}\right),z||<\frac{\varepsilon }{3}, \end{array}$$

for every *z* ∈ *X*. Now write *n*_0_ = max⁡{*n*_1_, *n*_2_}. Thus, for *r* ≥ *n*_0_, we have

(13) 1hr∑k∈Ir||f(xk+1)−f(xk),z|| ≤1hr∑k∈Irf||(xk+1)−fn1(xk+1),z||  +1hr∑k∈Ir||fn1(xk+1)−fn1(xk),z||  +1hr∑k∈Ir||fn1(xk)−f(xk),z|| <ε3+ε3+ε3=ε.

Hence,

(14) $$\begin{array}{c} \underset{r\to \infty }{\lim}\frac{1}{h_{r}}\underset{k\in I_{r}}{\sum }||f\left(x_{k+1}\right)-f\left(x_{k}\right),z||=0. \end{array}$$

Thus *f* preserves *N*_*θ*_-quasi-Cauchy sequences. This completes the proof of the theorem.

## 4. Conclusion

In this paper, we investigate strongly lacunary continuity and some other kinds of continuities defined via a lacunary sequence and we prove interesting theorems related to these kinds of continuities. The results in this paper are extensively deeper than existing related results in the literature. We note that the notion of a strongly lacunary quasi-Cauchy sequence coincides with the notion of a strongly lacunary convergent sequence in a complete non-Archimedean 2-normed space, and so the set of strongly lacunary ward continuous functions coincides with the set of strongly lacunary sequentially continuous functions in a complete non-Archimedean 2-normed space (see [21] for the related concepts in an ultrametric field). For a further study, we suggest to investigate strongly lacunary quasi-Cauchy sequences of points for the fuzzy functions in a 2-normed fuzzy spaces. However, due to the change in settings, the definitions and methods of proofs will not always be analogous to those of the present work (see [22, 23] for the definitions and related concept in fuzzy setting). We note that the study in this paper can be generalized to *n*-normed spaces as another further study.
